# Colour Morphs as Alternative Solutions to the Trade‐Off Predicted by the Immuno‐Competence Handicap Hypothesis

**DOI:** 10.1111/1749-4877.13028

**Published:** 2025-08-11

**Authors:** Roberto Sacchi, Alan J. Coladonato, Stefano Scali, Marco A. L. Zuffi, Rupert Palme, Marco Mangiacotti

**Affiliations:** ^1^ Department of Earth and Environmental Science University of Pavia Pavia Italy; ^2^ Museo di Storia Naturale di Milano Milano Italy; ^3^ Museo di Storia Naturale dell'Università di Pisa Calci PI Italy; ^4^ Experimental Endocrinology, Department of Biomedical Sciences University of Veterinary Medicine Vienna Austria

**Keywords:** aggressive behaviour, colour morphs, immune‐competence, polymorphism, trade‐off

## Abstract

Colour morphs in polymorphic species are associated with a suite of heritable traits governed by distinct genetic loci, each corresponding to alternative fitness peaks. Hormonal pleiotropy has been proposed as a mechanism maintaining these morphs, though experimental evidence is lacking. In this study, we tested whether white and yellow morphs of the common wall lizard (*Podarcis muralis*) adopt alternative strategies shaped by the immuno‐competence handicap hypotheses (ICHH). Specifically, we experimentally elevated plasma testosterone levels via transdermal administration and measured changes in (i) immune response using phytohaemagglutinin (PHA) swelling and (ii) the aggressive behaviour in response to a mirror test simulating territorial intrusion. We found that testosterone‐induced immune suppression was stronger in white males, while aggression decreased in both morphs. Elevated testosterone eliminated the baseline differences between morphs in both immunity and aggression. These findings provide the first experimental support for morph‐specific life‐history strategies in common wall lizards, consistent with the ICHH: Yellow males prioritize aggression over immunity, while white males invest more in survival at the cost of competitiveness.

## Graphical abstract

In this study, we tested whether white and yellow morphs of the common wall lizard (*Podarcis muralis*) adopt alternative strategies shaped by the immuno‐competence handicap hypotheses (ICHH). We found that testosterone‐induced immune suppression was stronger in white males, while aggression decreased in both morphs. Elevated testosterone eliminated the baseline differences between morphs in both immunity and aggression.

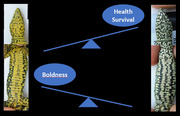

## 1 Introduction

Understanding the origin of colour polymorphism—that is, the stable co‐occurrence of genetically inherited colour variants (morphs) within the same population (Ford 1945; Huxley 1955)—remains a central challenge in evolutionary ecology. Morphs are typically interpreted as alternative peaks in the fitness surfaces, maintained by disruptive selection in response to specific life‐history trade‐offs (Sinervo and Lively 1996; Svensson et al. 2001; McKinnon and Pierotti 2010). Each morph represents a distinct combination of heritable traits governed by separate sets of loci, including those controlling colour, which interactively contribute to fitness (Whitlock et al. 1995; Sinervo and Svensson 2002). These favourable genetic correlations are shaped by correlative selection, which favours linkage disequilibrium between loci responsible for adaptive trait combinations that are identifiable as morphs (Sinervo and Svensson 2002). There is general agreement that correlational selection is the first step in building up the adaptive genetic associations (Svensson 2017), but other selective forces, such as frequency‐dependent selection, are thought necessary to maintain allele combinations within morphs over time (Sinervo and Lively 1996; Sinervo and Svensson 2002).

Pleiotropic effects of the colour loci may also contribute to morph maintenance. Hormones, for instance, can affect multiple traits including colour, behaviours, physiology and reproductive traits (Sinervo et al. 2000; Comendant et al. 2003; Barron et al. 2015). Hormonal changes modulate behavioural responses, which make individuals able to deal with environmental stressors (e.g., glucocorticoids) or sexual competition (e.g., testosterone). On the other hand, persistently elevated hormone levels can have detrimental effects, such as reduced immune competence (Munck et al. 1984; Kurtz et al. 2007). This sets a trade‐off between immune function and other traits relevant for individual survival and reproduction, ultimately affecting the individual fitness (Folstad and Karter 1992). Because individuals vary in how they respond to stressors and how behaviour and physiology adjust to these stressors, frequency‐dependent selection may act on morph‐specific strategies to balance these trade‐offs (Comendant et al. 2003).

In this study, we tested the role of hormones in maintaining the colour morphs of the common wall lizard (*Podarcis muralis*) by experimentally manipulating plasma testosterone (T) levels. This species is a small diurnal lizard widespread in central and southern Europe (Sillero et al. 2014), which exhibits three discrete colour morphs (yellow, white and red) genetically determined in both sexes (Sacchi et al. 2007b; Andrade et al. 2019). However, the colour variation in the species is complex, and also intermediate morph phenotypes (white‐yellow, white‐red and yellow‐red) frequently occur (Sacchi et al. 2013). Morphs are associated with two small regulatory regions near genes associated with pteridine (SPR locus) and carotenoid (BCO2 locus) metabolism. Red colouration is conferred by a recessive allele at the SPR locus, whereas the presence the yellow colouration is due to a recessive allele at the BCO2 locus (Andrade et al. 2019). Mating is not assortative based on colour morphs (Sacchi et al. 2015). Over recent decades, morph‐specific patterns in several life‐history traits have been described in *P. muralis* (Calsbeek et al. 2010; Galeotti et al. 2013; Pérez i de Lanuza et al. 2013; Scali et al. 2013, Sacchi et al. 2015; Mangiacotti et al. 2019). Notably, yellow males are immunosuppressed compared to the other morphs, as determined by subcutaneous injection of phytohaemagglutinin (PHA) (Sacchi et al. 2007a, 2017a). Testosterone is known to suppress immune function (Oppliger et al. 2004), potentially reducing survival, even though data on morph‐specific survival are lacking. Testosterone levels in males peak at the start of the mating season and then decline—a pattern documented in other lizards as well. While average T levels do not differ significantly between morphs, yellow males maintain significantly higher levels early in the season, followed by a steeper decline compared to white and red males (Sacchi et al. 2017b). The seasonal trends of the hormone align with the seasonal shifts in the aggressive behaviour (Coladonato et al. 2020). Yellow males are more aggressive than white males early in the breeding season, while the white males become more aggressive later on (Sacchi et al. 2017a; Coladonato et al. 2020). Females of this species typically produce up to three clutches per year (Sacchi et al. 2012), with clutch size typically declining as the reproductive season progresses (Barbault and Mou 1988; Castilla and Bauwens 2000b). Furthermore, later clutches are also more variable (and more unpredictable) in size, as the resources allocated in the second reproduction derive from recent food intake, and not from fat reserves as in the first one (Braña et al. 1992). Thus, males investing more in reproduction early in the season might gain a reproductive advantage compared to males delaying reproduction when females lay second (or third) clutches. Nevertheless, direct evidence of morph‐specific differences in breeding success in males remains lacking.

Based on these observations, we hypothesized that white and yellow males follow two distinct, alternative strategies shaped by the trade‐off outlined in the immuno‐competence handicap hypotheses (ICHH; Folstad and Karter 1992). High testosterone plasma levels can suppress immune function, favour parasite infections, stimulate risky behaviours, and ultimately reduce survival (Olsson et al. 2000; Klukowski and Nelson 2001; Cox and John‐Alder 2007). Therefore, it may be impossible for males to simultaneously maximize both stamina and aggressiveness. Accordingly, yellow males may follow a “risky” strategy, prioritizing territorial aggression at the expense of a reduced immune function, whereas white and red males may follow a “conservative” strategy, favouring survival over direct competition. In this context, colour morphs act as status badges, signalling individual strategy to conspecifics.

The effect of experimental T levels manipulation is well documented in many lizard species: Treated males obtain larger and higher‐quality home ranges, and are more successful in fighting against opponents (Marler and Moore 1989; DeNardo and Sinervo 1994). On the other hand, increasing plasma T levels leads to a decrease in cell‐mediated immunity (Olsson et al. 2000; Oppliger et al. 2004). Although T manipulation has been successfully tested in *Podarcis* lizards (Oppliger et al. 2004; Baeckens and Van Damme 2018), morph‐specific responses have not been previously detected. In the present study, we experimentally elevated plasma T levels in yellow and white males of the common wall lizard to test whether morphs differ in their physiological and behavioural responses to this hormonal trade‐off outlined by the ICHH. If yellow males adopt the risky strategy and white males the conservative one, we expect morph‐specific responses of testosterone on both immune function and aggressive response. Specifically, we predicted that immune suppression would be more pronounced in white males (who prioritize survival) and that aggressive behaviour would decline more sharply in white than in yellow males.

## 2 Materials and Methods

### 2.1 Lizard Collection and Housing

Forty adult males of common wall lizards were captured during March 2019 (20–26) in and around the town of Pavia (Northern Italy, 45°11′N, 9°9′E). Only pure white (*n* = 20) and yellow (*n* = 20) morphs were collected, since white and red males show the same T level seasonal pattern (Sacchi et al. 2017a), and red males occur at low frequency in Pavia populations (Sacchi et al. 2007b). The body size (snout–vent length, SVL) of each individual was measured to the nearest 0.5 mm and weighed to the nearest 0.1 g. We individually housed lizards in 20 × 30 × 20 cm transparent plastic boxes, with a brick as shelter/basking site, a small bowl with water, a UVB 5% lamp turned on 8 h/day (starting from 8:00 a.m., Sylvania F30W Reptistar T8 UVB 5%), and heating pads as hot spot. We provided mealworms as food (one mealworm/day), which were dusted with vitamin and calcium supplements two times for week. A minimum acclimation period of 1 week was given before starting trials, and we released all lizards at their capture sites following experimental trials. No lizard was injured or killed during the study, and all lizards looked healthy at release.

### 2.2 Manipulation of Testosterone

Plasma T levels were manipulated according to the non‐invasive technique already proposed for lacertid species (Knapp and Moore 1997; Belliure et al. 2004; Oppliger et al. 2004), and successfully used in *P. muralis* (Baeckens et al. 2016). Following this last study, we increased plasma T levels—while maintaining levels within the species’ estimated physiological range—through transdermal administration of a mixture of the steroid hormone and sesame oil to the lizard's dorsal skin. Lipophilic molecules such as testosterone are able to pass through lizard scales into bloodstream (Baeckens et al. 2016). The administered T‐dose was the same as in Baeckens et al. (2016), obtained by diluting testosterone (4‐androsten‐17b‐ol‐3‐one; Sigma #86500) in commercial sesame oil to a concentration of 4 µg/µL. Males received 4 µL of the hormone dilution every 2 days over 4 consecutive weeks. We applied a droplet of the hormone solution on the back of lizards early in the morning, before turning on the UVB lamp and heating pad, when individuals were not in activity, to minimize the stress. To ensure that the treatment successfully increased plasma T levels, we used a non‐invasive steroid analysis based on faecal droppings (Palme 2005; Palme et al. 2013). We collected faecal drops for each individual before the hormonal treatment and at the end of the experiment. Samples were frozen and stored in individual labelled Eppendorf tubes (0.5 mL) at −20°C. Concentrations of faecal testosterone metabolites (TMs) were measured with a testosterone enzyme immunoassay (EIA), previously utilized in lizards' droppings (Baeckens et al. 2016). For details of the EIA, see Auer et al. (2020).

### 2.3 Behavioural Experiments

Each individual performed double behavioural experiments before and after the hormone treatment. We measured the aggressive response of a focal male by the introduction of a small mirror (15 × 15 cm) into the plastic enclosure to mimic the intrusion of a stranger male into its own territory. We had previously shown that common wall lizards perceive their own mirror image as a rival, and behave aggressively in response (Scali et al. 2019; Coladonato et al. 2020; Sacchi et al. 2021). This procedure allows the experimenters to control for the effects of differences in size and motivation between opponents on the aggressive response of the focal male, since the mirror image exhibits the same size and motivation as the male (Sacchi et al. 2021). Before starting a trial, we removed the water bowl and put a partition dividing the arena into two halves. We then placed the mirror at the far end of the half without the lizard. After 5 min, we assumed focal lizards had habituated to disturbance and removed the partition, thereby allowing the lizard to interact with the mirror. To avoid visual disturbance during the trials, the four sides of the arena were externally covered by opaque, white plastic panels. Before each trial, the male was heated for 2 min using a 75 W halogen infra‐red lamp (Reptiles‐Planet.com) positioned 40 cm above the arena. After switching off the lamp, the mean (± SD) body temperature of males just before starting the trial (measured with a handheld infra‐red thermometer Lafayette TRP‐39, Lafayette Instrument Co., Lafayette, Indiana, USA; sensitivity: 0.1°C; precision: ± 2%) was 37.6 ± 1.8°C. The lizard movements were recorded using a webcam (Microsoft LifeCam HD 3000) mounted on an easel, 60 cm above the arena, and connected to a laptop by a 3‐m cable. Recording was managed by Free2X software v1.0.0.1 (freely available at: http://www.free2x.com/webcam‐recorder/), setting quality to 800 × 600 pixels and 15 frames per second (fps). Recording duration was set to 15 min, following the first lizard movement (i.e., tongue flicking, head movement, etc.). Room temperature was set to 28°C to reduce thermal loss during the trials. Trials took place between 10 a.m. and 2 p.m., and the order of morphs was randomized to control for potential effects of daytime. We repeated the trial the subsequent day if the lizard did not move after 10 min from the start. The first series of trials was performed between 3 and 18 April and the second series between 8 and 23 June.

### 2.4 Response Variables

We used BORIS (Friard and Gamba 2016) to analyse videos and extract four response variables. The first three variables were used to assess the aggressive behaviour responses (Coladonato et al. 2020; Ficetola et al. 2021): (i) the number of times that lizards entered in the half of the enclosure containing the mirror (*N*_mirror_), (ii) the time (seconds) spent in the half of the enclosure with the mirror (Time) and (iii) the total number of bites against the mirrored image (Bites). These three variables can be interpreted as increasing levels in a rank of aggression (see Sacchi et al. 2021 for details). The fourth variable was the tongue flick rate (TF, number of flicks divided by Time) measured in the half of the enclosure containing the mirror. This variable evaluated the basal explorative behaviour of each individual when facing a potential contestant (Sacchi et al. 2021). For simplicity, we hereafter refer to Bites, Time and *N*_mirror_ as forms of ‘aggressive behaviour’, and to TF as ‘exploratory behaviour’. All response variables achieved normality (Bites required a log‐transformation), and were weakly correlated with each other (Spearman correlation coefficient: |*r_s_* |< 0.52).

### 2.5 Immune‐Response Test

*In vitro* activation of lymphocytes enabled us to repeatedly challenge the immune system of the same individual, preventing the adaptive immune response from forming an immunological memory (Sacchi et al. 2017a). We used it to evaluate the change in the immune function of each individual in response to the hormonal treatment (i.e., before and after the treatment). Blood samples (20 µL) were collected in heparinized capillary tubes from the post‐orbital sinus (MacLean et al. 1973) and inoculated in 15 mL of RPMI 1640 medium supplemented with 10% bovine serum. Then, we divided the cell suspension into two 7 mL sub‐cultures, one of which was inoculated with 1% PHA solution (PHA‐P Sigma, 50 mg in 10 mL of phosphate‐buffered saline; Oppliger et al. 2004; Sacchi et al. 2017a). The last 1 mL of solution was used to assess starting lymphocyte concentration using a Neubauer chamber. Each sub‐culture was then distributed in two 1.5 mL culture tubes and incubated at 32°C for 3 days. Afterward, cells were collected, re‐suspended and newly counted. This second count involved only proliferating lymphocytes. Colony‐forming units (CFU) and the total T‐cells per mL to the corresponding were assessed, and stimulation was evaluated by the fold‐change of the PHA sample with respect to the control (Sacchi et al. 2017a).

### 2.6 Statistical Analysis

First of all, we verified whether the treatment has actually increased the faecal T concentration using a random intercept linear mixed‐effects model (LMM), in which the fixed effects were the treatment (pre and post), the morph and their interaction. The individual entered the model as a random effect.

Second, to examine if the aggressive response of lizards changed according to the hormonal treatment, we still used LMMs, one for each response variable. Fixed effects were the hormonal treatment, the morph and their interaction to account for possible differential effects of treatment due to colour morphs. We also added SVL (after standardization to zero‐mean and unit variance) as a fixed effect to control for the possible confounding effect due to individual size. The individuals entered the model as a random effect. Bites showed a Poisson‐like distribution with overdispersion (sd/mean = 29) and zero inflation, while the other variables assumed a normal distribution (one‐sample Kolmogorov–Smirnov test, all *p* values larger than 0.05). Thus, we ran a zero‐inflated negative binomial regression for bites and LMM via Satterthwaite's degrees of freedom for the other variables.

Third, we used the same approach to search for morph‐specific changes in the immune response according to the hormonal treatment. Lymphocytes and CFU were the dependent variables in two separate LMMs, in which the fixed and the random components were the same as in the analyses of the aggressive response. Both dependent variables followed a normal distribution and were not transformed.

Finally, we tested for the effects of the hormone treatment on the relationship between aggressive behaviour and immune‐responsiveness through random intercept LMMs where Bites, Time, *N*_mirror_ and TF were the dependent variables, each in a separate model, and the three‐way interaction morph × treatment × immunity was the fixed effect. Immunity was, in turn, represented by the total lymphocytes or CFU counts. Therefore, eight models were prepared, with individual ID as a random effect on the intercept.

LMMs were fit in a Bayesian analytical framework available in the package JAGS 4.3.0 (http://mcmc‐jags.sourceforge.net/), using flat priors for coefficients and intercept (μ = 0 and σ = 0.001), and uninformative half‐Cauchy priors (x0 = 0, γ = 25) for both σ^2^_error_ and σ^2^_individual_. For all models, Markov Chain Monte Carlo parameters were set as follows: number of independent chains = 3; number of iterations = 34 000; burning = 4000; thinning = 3. We checked convergence through trace plot and autocorrelation along chains, and results from the posterior distribution are reported as the half sample mode (HSM, Bickel and Frühwirth 2006) with 95% and 50% highest density intervals (HDI95, Kruschke 2010). All analyses were done in R 3.6.1 (R Core Team 2022) using the packages R2jags (Su and Yajima 2015), modest (Poncet 2012) and HDInterval (Meredith and Kruschke 2018).

## 3 Results

One white male was excluded from the experiments because it still had not eaten after 1 week of acclimation, and was therefore released. Faecal samples from five lizards (1 white and 4 yellow individuals) were not heavy enough for steroid analysis and were discarded. Consequently, the final sample included 34 individuals (18 white and 16 yellow).

### 3.1 Hormonal Treatment

Following the four treatment weeks, faecal TM concentrations doubled compared to the initial values in all males of both morphs (for both morphs: *P*_post‐pre>0_ = 0.999, Table 1). No difference between morphs were observed before (*P*_white>yellow_ = 0.305), whereas after treatment yellow males had higher faecal TM concentration (*P*_white>yellow_ = 0.011, Table 1).

**TABLE 1:** Posterior distributions of testosterone metabolite concentration (ng/g) in faecal pellets of white and yellow morphs before and after the hormonal treatment (see Methods section for details). HSM and HDI95 estimates (in brackets) are shown.

	Pre‐treatment	Post‐treatment	Difference (post‐pre)
White (*n* = 18)	279 (170–384)	479 (376–582)	+73.5% (51.6–121.1) *P*_d>0_ = 0.999
Yellow (*n* = 16)	315 (188–440)	626 (500–743)	+98.7% (68.9–165.5) *P*_d>0_ = 0.999
Difference (white‐yellow)	−39 (−165 to 89) *P*_whi>ylw_ = 0.305	−145 (−251 to −38) *P*_whi>ylw_ = 0.011	

### 3.2 Behavioural Response and Hormonal Treatment

In 65 out of 68 trials (95.5%), lizards approached the mirror, and 33 of them (50.8%) bit the reflected image. Focal lizards entered the half portion of the cage hosting the mirror (*N*_mirror_) on average 7.3 ± 4.7 (range 0–19) times, whereas the time spent in the half‐mirrored cage (Time) ranged from 0 to 866 s, being on average 417 ± 274 s. The mean number of tongue flicks (TF) was 43 ± 40 (0–196).

**FIGURE 1:**
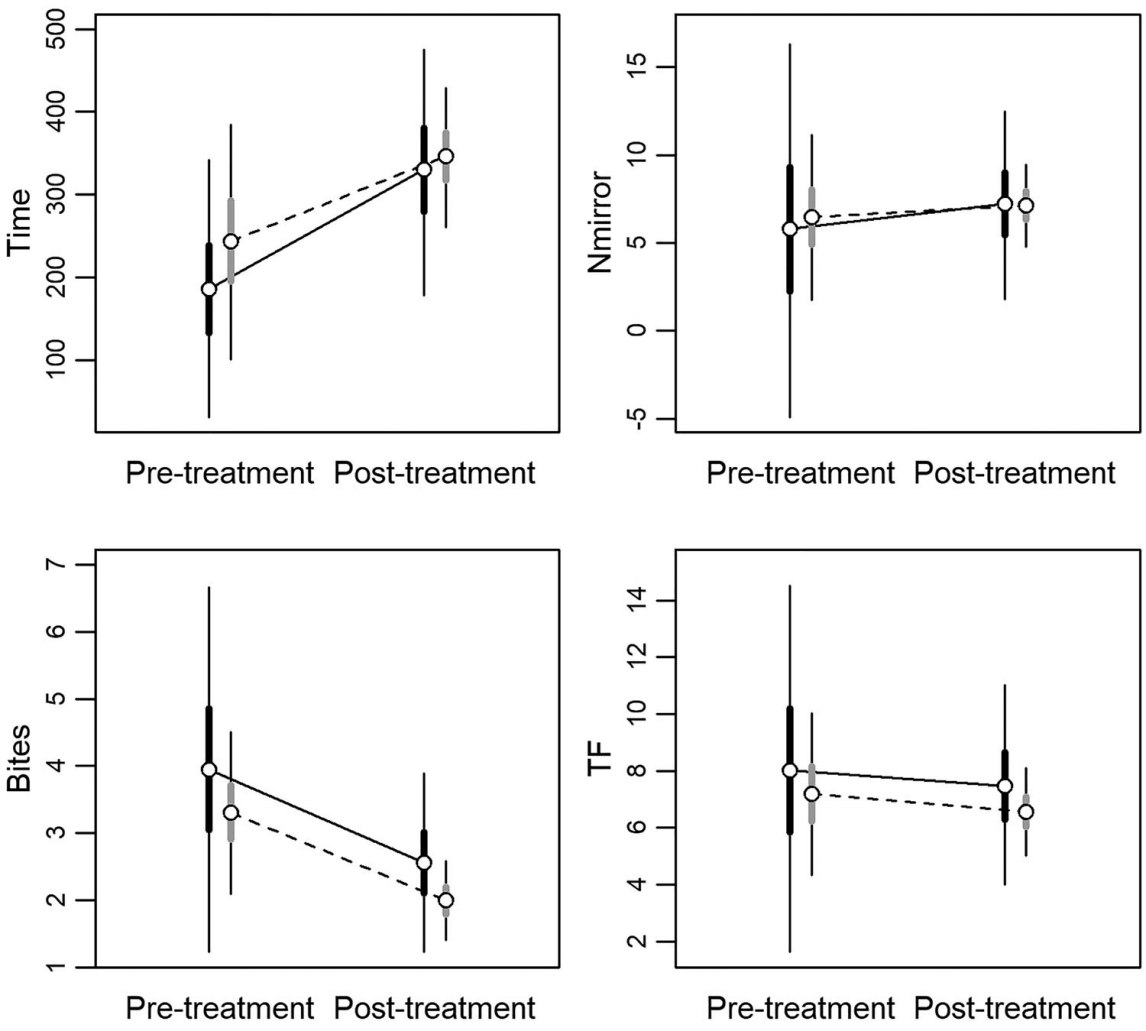
Bayesian model predictions for the aggressive and exploratory responses of common wall lizards before (pre‐treatment) and following (post‐treatment) the testosterone supplementation according to morph (*black and grey bars* are for white and yellow morphs, respectively). *Time*: time in seconds spent in the half enclosure containing the mirror; *N*_mirror_: the number of times the lizard entered the half of the arena containing the mirror; *Bites*: the total number of bites against the mirror image (log‐transformed, see text for details); *TF*: the number of tongue flicks in the half of the arena containing the mirror. Circles indicate HSM, and thick and thin lines represent HDI_50_ and HDI_95_, respectively.

Following the hormonal treatment, both white and yellow morphs increased the time spent in the half‐mirrored cage (Figure 1), but white males responded more than yellow ones (relative increase, white morph: +77.4%, yellow morph: +41.8%; Table 2). Consequently, the extent of the difference between morphs observed before the hormonal treatment decreased in the post‐treatment, when white and yellow morphs showed very similar values (Table 2 and Figure 1). The number of bites followed the opposite pattern (Figure 1), and in both morphs, bites decreased sensibly after the hormonal treatment (Table 2). Further, the extent of the decrease was similar in both morphs (white morph: −35.2%; yellow morph: −39.6%), so white males bite the mirrored image more than yellow ones irrespective of the hormone concentration. *N*_mirror_ did not appreciably vary between the two measurements within a morph (Figure 1), and the same occurred for the comparison between morphs in each measurement (Table 2). The TF also did not appreciably change following the hormonal treatment (Table 2 and Figure 1).

**TABLE 2:** Posterior distributions for the aggressive and exploratory responses of common wall lizards before (pre‐treatment) and following (post‐treatment) the testosterone supplementation according to morph. HSM and HDI95 estimates (between brackets) are shown.

Variable		Pre‐treatment	Post‐treatment	Difference (post‐pre)
Time	White	186 (55–316)	330 (204–451)	+144 (40–247) *P*_d>0_ = 0.998
	Yellow	244 (124–363)	346 (275–416)	+102 (23–181) *P*_d>0_ = 0.984
	Difference	58 (−45 to 161) *P*_ylw>whi_ = 0.825	16 (−65 to 101) *P*_ylw>whi_ = 0.622	
*N*_mirror_	White	5.82 (−3.09 to 14.4)	7.22 (2.73–11.6)	+1.41 (−3.91 to 6.78) *P*_d>0_ = 0.670
	Yellow	6.46 (2.53–10.4)	7.12 (5.15–9.06)	+0.67 (−2.90 to 2.81) *P*_d>0_ = 0.685
	Difference	0.64 (−4.94 to 6.34) *P*_ylw>whi_ = 0.578	−0.10 (−2.90 to 2.81) *P*_ylw>whi_ = 0.481	
Bites	White	3.95 (1.70–6.19)	2.56 (1.45–3.67)	−1.39 (−2.77 to −0.02) *P*_d>0_ = 0.048
	Yellow	3.31 (2.30–4.29)	2.00 (1.50–2.48)	−1.31 (−1.92 to −0.69) *P*_d>0_ < 0.001
	Difference	−0.64 (−2.08 to 0.83) *P*_ylw>whi_ = 0.229	−0.57 (−1.28 to 0.15) *P*_ylw>whi_ = 0.093	
TF	White	8.02 (2.65–13.4)	7.48 (4.58–10.4)	−0.55 (−3.72 to 2.66) *P*_d>0_ = 0.385
	Yellow	7.19 (4.82–9.55)	6.55 (5.27–7.84)	−0.63 (−2.05 to 0.79) *P*_d>0_ = 0.228
	Difference	−0.85 (−4.28 to 2.63) *P*_ylw>whi_ = 0.343	−0.91 (−2.81 to 0.96) *P*_ylw>whi_ = 0.207	

### 3.3 Immune Response and Hormonal Treatment

**FIGURE 2:**
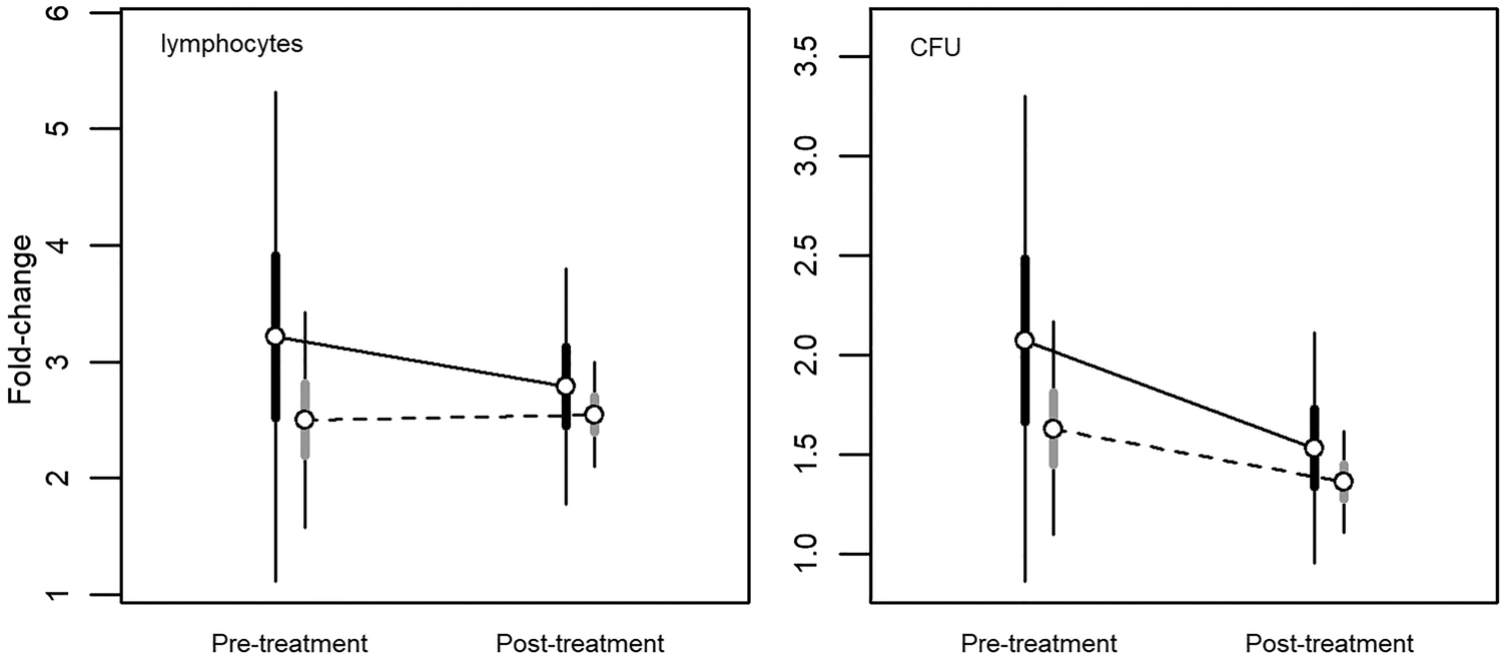
Bayesian model predictions for the immune response of common wall lizards before (pre‐treatment) and following (post‐treatment) the testosterone supplementation according to morph (*black and grey bars* are for white and yellow morphs, respectively). *Lymphocytes*: the number of proliferating lymphocytes after PHA inoculation; *CFU*: colony‐forming units following PHA inoculation (see text for details). Circles indicate HSM, and thick and thin lines represent HDI_50_ and HDI_95_, respectively.

The PHA injection actually stimulated the lizards’ immune system in pre‐ and post‐hormonal treatment, as the fold‐changes (Table 3) were systematically higher than one in all cases for both the total lymphocyte count and the colony‐forming units (CFU; *P*_fold‐change>1_> 0.959). According to lymphocyte values, white males showed higher immune competence than yellow ones, both before and after the hormonal treatment (Table 3), even if the difference was reduced by two‐thirds following the testosterone administration (Table 3 and Figure 2). This reduction in the difference between morphs was entirely due to white males, which showed an evident immunosuppression due to the hormonal treatment (Figure 2). Compared to the initial values, the immune response of white males decreased by about 13% after testosterone administration (Table 3), whereas the immune response of yellow males showed no change at all (Table 3).

**TABLE 3:** Posterior distributions for the immunological response of common wall lizards before (pre‐treatment) and following (post‐treatment) the testosterone supplementation according to morph. Values are fold‐changes with respect to the control. HSM and HDI95 estimates (between brackets) are shown.

Variable		Pre‐treatment	Post‐treatment	Difference (post‐pre)
Lymphocytes	White	3.22 (1.48–4.97)	2.79 (1.94–3.63)	−0.43 (−1.50 to 0.653) *P*_d<0_ = 0.749
	Yellow	2.50 (1.74–3.27)	2.55 (2.17–2.92)	+0.04 (−0.43 to 0.52) *P*_d<0_ = 0.437
	**Difference (yellow‐white)**	−0.71 (−1.84 to 0.40) *P*_ylw<whi_ = 0.855	−0.24 (−0.78 to 0.31) *P*_ylw<whi_ = 0.769	
CFU	White	2.07 (1.05–3.09)	1.53 (1.05–2.02)	−0.54 (−1.17 to 0.09) *P*_d<0_ = 0.922
	Yellow	1.63 (1.18–2.08)	1.36 (1.15–1.58)	−0.27 (−0.55 to 0.01) *P*_d<0_ = 0.945
	**Difference (yellow‐white)**	−0.45 (−1.11 to 0.21) *P*_ylw<whi_ = 0.869	−0.17 (−0.49 to 0.14) *P*_ylw<whi_ = 0.818	

The CFU followed the same pattern of response as observed for total lymphocytes (Table 3). White males had higher immune response than yellow males in both measurements (Table 3), but the difference was less marked (about half) after testosterone administration (Table 3 and Figure 2). Differently from total lymphocyte count, both morphs showed immune‐suppression in response to testosterone administration, as the CFU sensibly decreased in the second measurement (Table 3 and Figure 2). However, the immune‐suppressive effect was about twice in white than in yellow males (Table 3).

### 3.4 Behavioural Response and Immune Function

**FIGURE 3:**
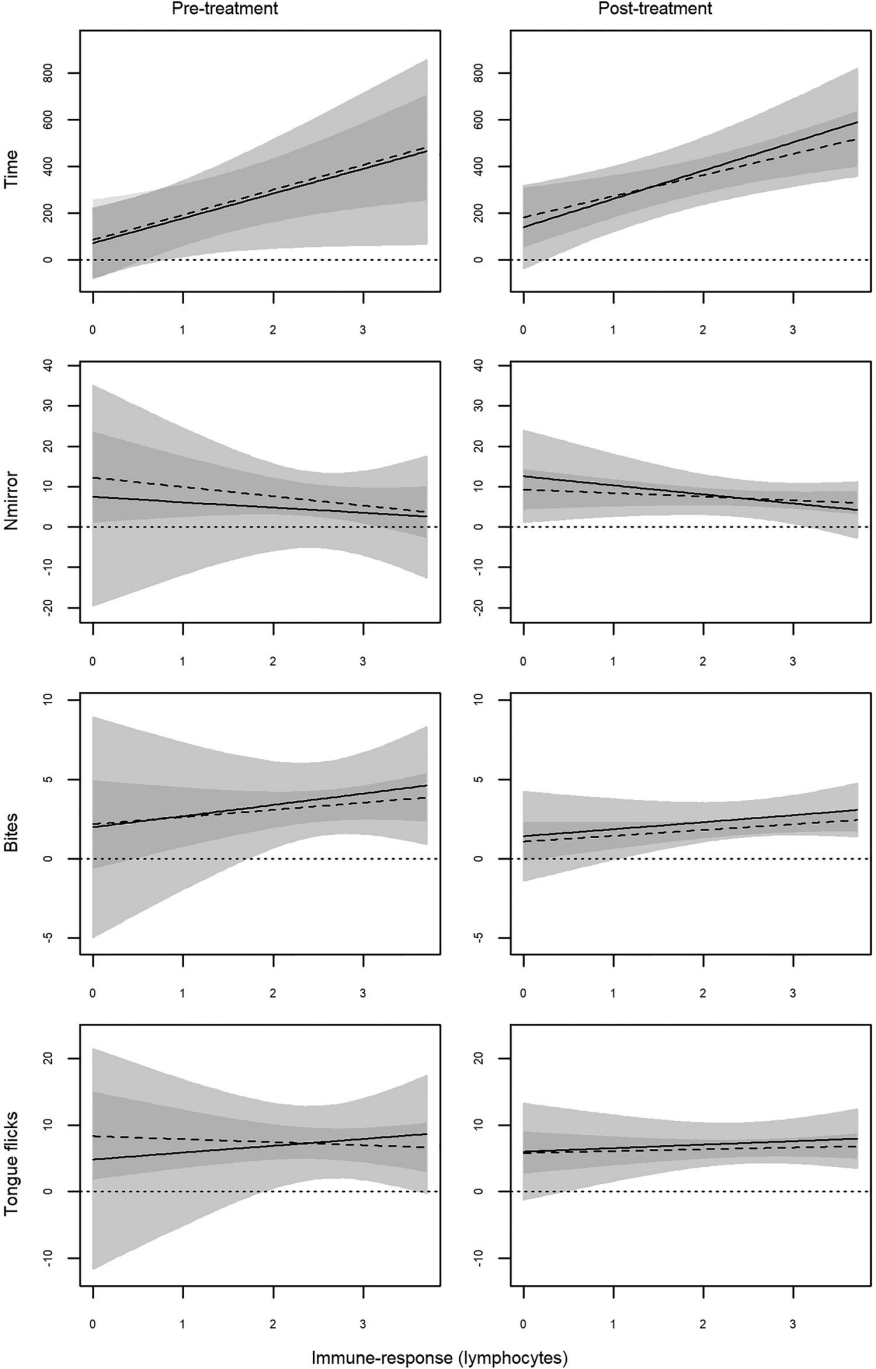
Bayesian model predictions for the relationship between behavioural response and immune‐response (total lymphocyte count) of common wall lizards before (pre‐treatment) and following (post‐treatment) the testosterone supplementation according to morph (solid and light grey are for white morph and dashed and dark grey are for yellow morph). Lines indicate HSM, and areas represent HDI95.

**FIGURE 4:**
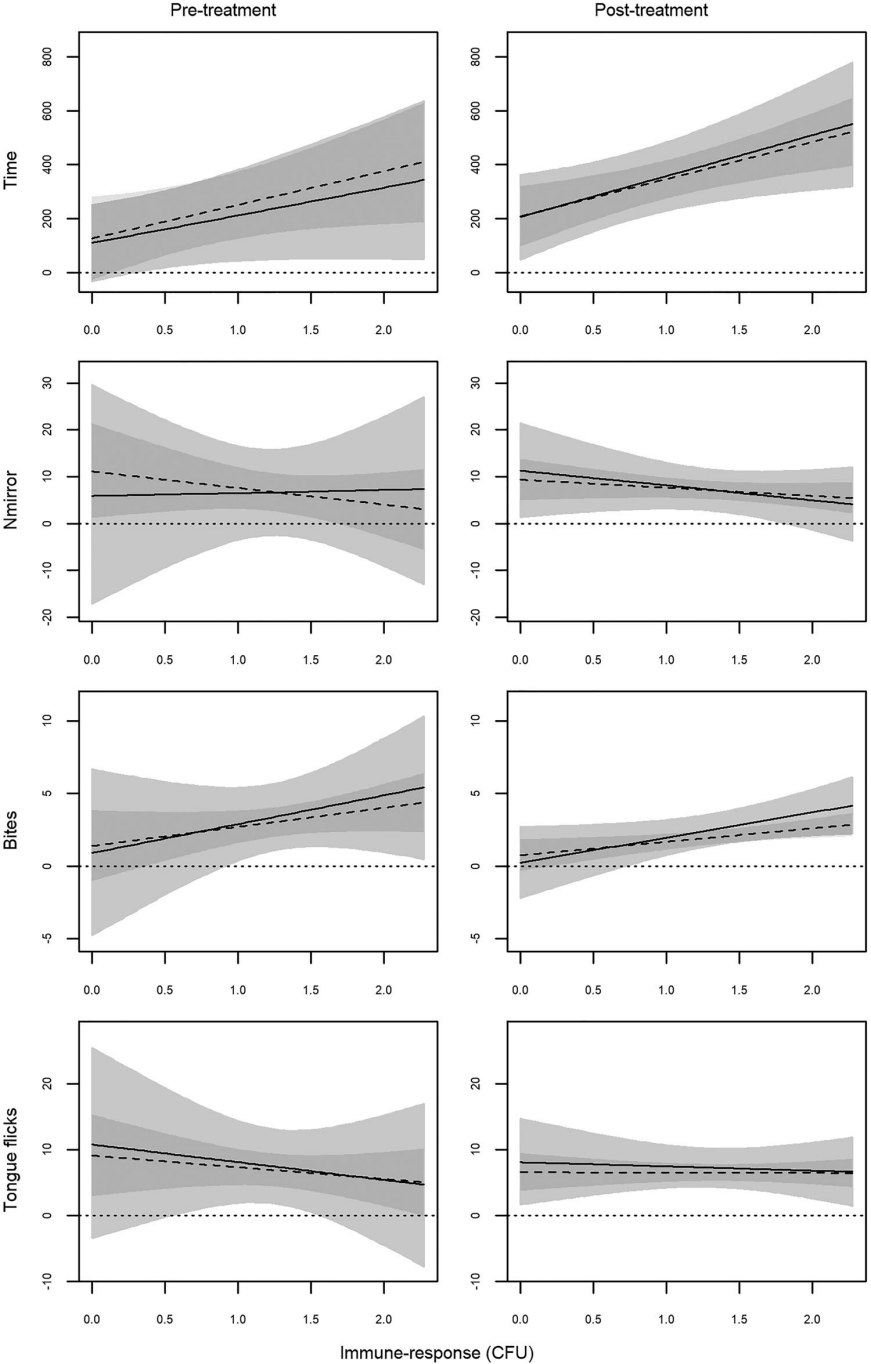
Bayesian model predictions for the relationship between behavioural response and immune‐response (colony‐forming units, CFU) of common wall lizards before (pre‐treatment) and following (post‐treatment) the testosterone supplementation according to morph (solid and light grey are for white morph and dashed and dark grey are for yellow morph). Lines indicate HSM, and areas represent HDI95.

The behavioural response clearly correlated with the immune function in a similar way in both morphs, with males with higher immune response entering less frequently in the half of the enclosure with the mirror, but remaining there for longer times and biting the mirrored image more frequently (Figures 3 and 4). Accordingly, Time increased with increasing immune function for both total lymphocytes (Table 4) and CFU (Table 5), while the opposite occurred for *N*_mirror_, which in general decreased with increasing immune function (Tables 4 and 5). Further, males with higher immune function bit more frequently the mirrored image (Tables 4 and 5), and the effect was particularly evident for the CFU in the post‐treatment (Table 5).

**TABLE 4:** Posterior distributions for the slopes of the relationships between behavioural responses and immune‐function as estimated by the total lymphocytes in the two morphs of the common wall lizard before (pre‐treatment) and following (post‐treatment) the testosterone supplementation. HSM and HDI95 (between brackets) estimates are shown.

Variable		Pre‐treatment	Post‐treatment	Difference (post‐pre)
Time	White	105 (−3 to 213) *P*_β>0_ = 0.945	121 (39–203) *P*_β>0_ = 0.991	+16 (−61 to 93) *P*_d>0_ = 0.628
	Yellow	106 (25–187) *P*_β>0_ = 0.984	90 (38–143) *P*_β>0_ = 0.998	−16 (−75 to 43) *P*_d<0_ = 0.674
	**Difference (yellow‐white)**	+1 (−80 to 83) *P*_ylw>whi_ = 0.515	−30 (−91 to 31) *P*_ylw<whi_ = 0.792	
*N*_mirror_	White	−1.19 (−11.3 to 8.77) *P*_β<0_ = 0.547	−2.20 (−6.40 to 1.95) *P*_β<0_ = 0.816	−1.0 (−8.18 to 6.23) *P*_d<0_ = 0.589
	Yellow	−2.27 (−6.51 to 1.95) *P*_β<0_ = 0.806	−0.89 (−2.70 to 0.92) *P*_β<0_ = 0.789	1.39 (−1.41 to 4.19) *P*_d>0_ = 0.795
	**Difference (yellow‐white)**	−1.10 (−8.18 to 6.03) *P*_ylw<whi_ = 0.599	1.32 (−1.42 to 4.07) *P*_ylw>whi_ = 0.782	
Bites	White	0.70 (−1.82 to 3.25) *P*_β>0_ = 0.677	0.44 (−0.58 to 1.47) *P*_β>0_ = 0.761	−0.26 (−2.12 to 1.57) *P*_d<0_ = 0.592
	Yellow	0.44 (−0.57 to 1.47) *P*_β>0_ = 0.767	0.36 (−0.08 to 0.81) *P*_β>0_ = 0.909	−0.08 (−0.77 to 1.55) *P*_d<0_ = 0.582
	**Difference (yellow‐white)**	−0.26 (−2.09 to 1.55) *P*_ylw<whi_ = 0.589	−0.08 (−0.76 to 0.61) *P*_ylw<whi_ = 0.579	
TF	White	1.06 (−5.07 to 7.02) *P*_β>0_ = 0.617	0.56 (−2.15 to 3.12) *P*_β>0_ = 0.639	−0.52 (−4.82 to 3.85) *P*_d<0_ = 0.580
	Yellow	−0.46 (−2.92 to 1.94) *P*_β<0_ = 0.621	0.29 (5.27–7.84) *P*_β>0_ = 0.660	0.74 (−0.87 to 2.32) *P*_d>0_ = 0.778
	**Difference (yellow‐white)**	−1.54 (−5.79 to 2.89) *P*_ylw<whi_ = 0.723	−0.28 (−1.95 to 1.48) *P*_ylw<whi_ = 0.605	

**TABLE 5:** Posterior distributions for the slopes of the relationship between behavioural responses and immune‐function as estimated by the CFU in the two morphs of the common wall lizard before (pre‐treatment) and following (post‐treatment) the testosterone supplementation. HSM and HDI95 estimates (between brackets) are shown.

Variable		Pre‐treatment	Post‐treatment	Difference (post‐pre)
Time	White	102 (−25 to 228) *P*_β>0_ = 0.906	151 (22–279) *P*_β>0_ = 0.972	49 (−63 to 159) *P*_d>0_ = 0.764
	Yellow	124 (0.4–247) *P*_β>0_ = 0.951	137 (54–221) *P*_β>0_ = 0.996	13 (−86 to 114) *P*_d<0_ = 0.587
	**Difference (yellow‐white)**	22 (−84 to 126) *P*_ylw>whi_ = 0.638	−14 (−108 to 83) *P*_ylw<whi_ = 0.582	
*N*_mirror_	White	0.50 (−17.0 to 17.5) *P*_β>0_ = 0.519	−3.19 (−10.1 to 3.51) *P*_β<0_ = 0.781	−3.68 (−16.2 to 9.11) *P*_d<0_ = 0.684
	Yellow	−3.67 (−11.1 to 3.63) *P*_β<0_ = 0.795	−1.73 (−4.68 to 1.17) *P*_β<0_ = 0.837	1.95 (−3.34 to 7.33) *P*_d>0_ = 0.724
	**Difference (yellow‐white)**	−4.19 (−15.8 to 7.70) *P*_ylw<whi_ = 0.721	1.45 (−3.03 to 6.01) *P*_ylw>whi_ = 0.701	
Bites	White	1.94 (−2.26 to 6.29) *P*_β>0_ = 0.775	1.73 (0.06–3.40) *P*_β>0_ = 0.955	−0.22 (−3.43 to 2.88) *P*_d<0_ = 0.545
	Yellow	1.30 (−0.47 to 3.08) *P*_β>0_ = 0.767	0.92 (0.20–1.64) *P*_β>0_ = 0.983	−0.37 (−1.67 to 0.90) *P*_d<0_ = 0.684
	**Difference (yellow‐white)**	−0.64 (−3.67 to 2.31) *P*_ylw<whi_ = 0.684	−0.80 (−1.91 to 0.31) *P*_ylw<whi_ = 0.882	
TF	White	−2.66 (−13.4 to 7.88) *P*_β<0_ = 0.664	−0.57 (−5.06 to 3.81) *P*_β<0_ = 0.584	2.16 (−5.78 to 9.98) *P*_d>0_ = 0.672
	Yellow	−1.76 (−6.27 to 2.60) *P*_β<0_ = 0.746	−0.02 (−1.94 to 1.82) *P*_β<0_ = 0.508	1.74 (−1.43 to 4.92) *P*_d>0_ = 0.818
	**Difference (yellow‐white)**	0.91 (−6.48 to 8.40) *P*_ylw>whi_ = 0.581	0.53 (−2.39 to 3.54) *P*_ylw>whi_ = 0.616	

Morph‐specific effects of the testosterone administration on the relationship between aggressive behaviour and immune response were detected for both Time and *N*_mirror_. Indeed, the posterior probability of the three‐way interaction term of the models deviated from zero in both total lymphocytes and CFU for Time (*P*_β<0_ = 0.814 and *P*_β<0_ = 0.741, respectively) and *N*_mirror_ (*P*_β>0_ = 0.763; *P*_β>0_ = 0.848, respectively). In the pre‐treatment assessment, the relationship between the two behavioural responses and the immune function was more stringent (i.e., narrower CI_95%_) in the yellow morph, while the white morph had a greater variability (Figures 3 and 4, left columns). After the testosterone administration, the variability in the white morph (Figures 3 and 4, right columns) sensibly reduced, while no relevant changes occurred in the yellow one. Consequently, the dependence of the behavioural response on the condition of the immune system became equally stringent in the two morphs. We did not find any support for an equivalent three‐way interaction for Bites with both lymphocytes (*P*_β>0_ = 0.578) and CFU (*P*_β>0_ = 0.546). However, we detected an appreciable positive main effect of the immune function (lymphocytes: *P*_β>0_ = 0.677; CFU: *P*_β>0_ = 0.775), a negative main effect of the treatment and a two‐way interaction morph × immune‐function, the last two only for the CFU (*P*_β<0_ = 0.614 and *P*_β<0_ = 0.641, respectively). In summary, the slope of the relationship between Bites and the immune‐response is steeper in white with respect to yellow males, irrespective of the hormonal treatment (Figures 3 and 4). However, as for Time and *N*_mirror_, the Bites versus immune‐function relationship had a wider variance in white males than yellow ones, and this difference no longer persisted after the hormone administration (Figures 3 and 4).

Finally, the effects of the immune response on TF were contrasting and not fully coherent, if not opposite, when considering lymphocytes and CFU as immune response measures (Figures 3 and 4). Indeed, TF increased with increasing total lymphocytes (Table 3) but, conversely, decreased with increasing CFU (Table 4). Morph‐specific effect appeared also for TF, but limited to lymphocytes (*P*_β>0_ = 0.736; CFU: *P*_β>0_ = 0.544). The general effect of the hormone administration on the TF versus immune‐response relationship has been to reduce the difference in slope between morphs (Figure 3).

## 4 Discussion

In this paper, we manipulated plasma testosterone level (proven by TMs measured in droppings) to test if the white and yellow morphs of the common wall lizards represent two alternative and opposite solutions to the trade‐off between immunity and the expression of secondary sexual characters depicted by the ICCH (Folstad and Karter 1992). Previous correlative results (Sacchi et al. 2007a, 2017a, 2017b; Coladonato et al. 2020) led us to hypothesise that white morphs should follow a healthy strategy, allocating more resources to the immune system at the expense of aggression in the defence of the territory. Conversely, we expected yellow males to follow a risky strategy, by allocating more resources to aggressive behaviour aimed at territory defence at the expense of effectiveness in immune response. We obtained substantial, robust experimental support for it.

First, before the hormone administration, yellow males were immunosuppressed compared to the white ones, confirming morph‐specific immune‐response previously observed in the species (Sacchi et al. 2007a, 2017a). According to the ICHH, increased plasma testosterone levels caused immunosuppression, but the effect was clearly morph‐specific, being sensibly more severe in the white morph. Accordingly to the hypothesis of playing the healthy strategy, we showed white males not being able to cope with increased testosterone plasma levels. Their immune system crashes when hormone levels are artificially raised beyond the maximum level that the individual can express by adopting an allocation strategy in the trade‐off to maximize the efficiency in immune function. Conversely, yellow males suffered a reduced immune suppression as the trade‐off setting for a greater expression of secondary sexual characters (i.e., the risky strategy) has predisposed the immune system to better resist the immunosuppressive effects of increased plasma hormone levels. The administration of the hormone, therefore, has the overall effect of breaking the correlation between the allocations into the immune function and secondary sexual character, with the final outcome of making the immune response in the two morphs similar.

Second, the administration of testosterone also depressed the intensity of the behavioural response in the post‐treatment measurement, in a way that individuals became less aggressive than during the pre‐treatment. Following the increase in plasma testosterone levels, males of both morphs bit less (i.e., reduced direct aggression), but stayed longer in the half enclosure with the mirror (i.e., increased indirect aggression). In other words, the hormone administration did not change the propensity of the individuals to face the intruder, but it rather caused a switch from the direct aggressive behavioural mode, based on fighting (riskier in terms of physical damage or injuries), to the indirect aggression behavioural mode, based on the threat (with a reduced risky of damage). The hormonal effect on the aggressive response was still more evident in the white morph. Consequently, the differences in aggressive response between morphs disappeared following the hormonal treatment. Interestingly, the hormonal treatment did not have any relevant effects on the explorative behaviour (i.e., TF), supporting the strict relationship between aggressive behaviour and plasma testosterone levels. This conclusion is supported by previous correlative studies on morph‐specific seasonal patterns of testosterone plasma levels (Sacchi et al. 2017b) and aggressive behaviour (Coladonato et al. 2020) we performed on the same species. Indeed, yellow males had higher aggressive response than white males at the beginning of the season when testosterone plasma levels are also higher in yellow than white males.

Third, in both morphs, the aggressive response, whether direct or indirect, strictly depended on the immune function, irrespective of plasma testosterone levels. That is, only individuals in better condition were able to sustain the costs of the territorial defence, in both morphs. This still reinforces the idea that immunity and aggression are linked in a trade‐off, and also the idea that the differences in immune response and aggressive behaviour between white and yellow morphs previously detected at a correlative approach (Sacchi et al. 2007a, 2017b) are due to the evolution of alternative responses to the impossibility of simultaneously maximizing the two functions.

We therefore supplied experimental evidence of the existence of two morph‐specific strategies in common wall lizards, depending on the investment in territorial aggression or effective immune response. Nonetheless, this does not mean that yellow males are more aggressive than white ones in absolute terms, but in a relative sense. Indeed, we showed that mean reaction norms for immune response, aggressive behaviour, and plasma testosterone levels are similar between morphs, but it is the seasonal pattern that varies differently (Sacchi et al. 2017b, 2021; Coladonato et al. 2020). Yellow males maintain higher T levels and display more aggression at the beginning of the season, but display a stronger subsequent decline until having lower T levels and aggressive response than white males later in the season. Increased aggressive behaviour in the early part of the season means more clashes among individuals, at the cost of lower long‐term survival due to both lower immune response and higher predatory risk (Marler and Moore 1988; Sacchi et al. 2009), to the benefit of white males who choose the conservative strategy. On the other hand, increased immune function means longer survival at the cost of a reduced competitive ability for obtaining the best territories, to the benefit of yellow males who may achieve more mating attempts. Data available on life span in this as other *Podarcis* lizards are scarce, but generally agree to suggest that sexual maturity can be reached just after the first winter, and the mean life duration as adults does not normally exceed 2 to 4 years (Barbault and Mou 1988; Galán 2004; Biaggini et al. 2011; Altunışık et al. 2016). Unfortunately, data on differential survival between morphs are still totally lacking, but even though the reduction in survival due to T‐mediated immunosuppressive effects was small (i.e., only one breeding season), direct effects on fitness would still be occurring. From an evolutionary point of view, the yellow strategy could be seen as a way to obtain a high reproductive success but over a short time interval (lifespan), while the white strategy should be a way of achieving less reproductive success but over a longer time interval. If the overall balance in terms of fitness between the two alternatives is equivalent over long time intervals, the selection should keep the strategies in balance over time, without one prevailing over the other. Hence, understanding how the two strategies link the reproductive opportunities, and therefore the possibility of mating with females, is crucial to figure out how they evolved and persisted within the same population. Females in Mediterranean lizards normally lay up to two and often three annual clutches (the larger individuals), from early spring to late summer (*Podarcis ionica*, Chondropoulos and Lykakis 1983; *P. atrata*, Castilla and Bauwens 2000a; *P. lilfordi*, Castilla and Bauwens 2000b; *P. bocagei*, Galán 1997, as well as common wall lizards, Barbault and Mou 1988). In Northern Italy, the first clutch deposition of *P. muralis* occurs between late April and the beginning of May, while the second one occurs between late May and mid‐July (Sacchi et al. 2012). In common wall lizards, as in other Mediterranean species, clutch size typically declines as the reproductive season progresses (Barbault and Mou 1988; Castilla and Bauwens 2000b). This seasonal variation is induced by differences in the proximate source of the energy allocated to different annual clutches. Furthermore, yolk production for the first clutch mainly derives from fat reserves stored before hibernation, whereas the energy shunted to subsequent clutches derives from recent food intake (Braña et al. 1992). This makes the first clutch a more remunerative and predictable resource for males in terms of fitness compared with the second clutch, and even more so the third one. Therefore, being very aggressive and defending the best territories in the first part of the season allows yellow males access to the most profitable females, and therefore achieve the relative highest reproductive success within a single season. Nevertheless, the high breeding success pays the cost of a shorter life, which for *Podarcis* lizards means no more than two consecutive seasons (Barbault and Mou 1988; Altunışık et al. 2016). On the opposite, reducing the risk of damage or injury in the period of maximum competition and investing in longevity may actually force white males to focus on less profitable, and more unpredictable clutches, having in return an additional time compared with yellow males. Hence, the variability of clutches laid by females along the season combined with the impossibility for males to compete all over the reproductive season might be the primary evolutionary driver promoting the setting of the trade‐off inherent in ICHH toward the dual solution of a risky versus conservative strategy.

Adopting a different colour badge depending on strategy can help male morphs to efficiently recognise the strategy of rivals and modulate their own behaviour, reducing the risk of income in unnecessary and potentially dangerous fighting. The scenario we have just depicted above allows us to predict that for a given male, a rival adopting its same strategy is potentially riskier, compared to another one who adopts a different strategy. Many species of vertebrates show a morph‐specific aggressive response. For example, males of the cichlid fish direct more aggressive attempts toward similarly coloured opponents (Dijkstra et al. 2007), and similar outcomes have been reported for the polymorphic sparrow, *Zonotrichia albicollis* (Horton et al. 2012). Morph‐specific aggression also occurs in lizards as reported for the ornate tree lizard (*Urosaurus ornatus*), and the tawny dragon *Ctenophorus decresii* (Hover 1985; Yewers et al. 2016). Recently, we experimentally showed that aggression is more common during homomorphic than heteromorphic contests, also in common wall lizards, and such a kind of interaction controls the patterns of spatial distribution of morphs (Scali et al. 2021). By manipulating testosterone plasma levels, we supplied experimental evidence in support of the hypothesis that colour morphs in this species have evolved as a status badge to inform conspecifics about the strategies played by the signaller, and decrease unnecessary conflicts among different colour morphs.

## Ethics Statement

All the protocols have been authorized by Italian Environmental Ministry (Aut. Prot. PNM/000685 22/11/2018, valid for the three years 2019– 2021).
